# Point‐of‐sale promotion of breastmilk substitutes and commercially produced complementary foods in Cambodia, Nepal, Senegal and Tanzania

**DOI:** 10.1111/mcn.12272

**Published:** 2016-04-15

**Authors:** Mary Champeny, Catherine Pereira, Lara Sweet, Mengkheang Khin, Aminata Ndiaye Coly, Ndeye Yaga Sy Gueye, Indu Adhikary, Shrid Dhungel, Cecilia Makafu, Elizabeth Zehner, Sandra L. Huffman

**Affiliations:** ^1^ Helen Keller International Washington, DC. USA; ^2^ JB Consultancy Bryanston South Africa; ^3^ Helen Keller International Phnom Penh Cambodia; ^4^ Helen Keller International Dakar Senegal; ^5^ Helen Keller International Kathmandu Nepal; ^6^ Helen Keller International Dar es Salaam Tanzania; ^7^ Consultant to Helen Keller International

## Abstract

In order to assess the prevalence of point‐of‐sale promotions of infant and young child feeding products in Phnom Penh, Cambodia; Kathmandu Valley, Nepal; Dakar Department, Senegal; and Dar es Salaam, Tanzania, approximately 30 retail stores per site, 121 in total, were visited. Promotional activity for breastmilk substitutes (BMS) and commercially produced complementary foods in each site were recorded. Point‐of‐sale promotion of BMS occurred in approximately one‐third of sampled stores in Phnom Penh and Dakar Department but in 3.2% and 6.7% of stores in Kathmandu Valley and Dar es Salaam, respectively. Promotion of commercially produced complementary foods was highly prevalent in Dakar Department with half of stores having at least one promotion, while promotions for these products occurred in 10% or less of stores in the other three sites. While promotion of BMS in stores is legal in Senegal, it is prohibited in Cambodia without prior permission of the Ministry of Health/Ministry of Information and prohibited in both Nepal and Tanzania. Strengthening legislation in Senegal and enforcing regulations in Cambodia could help to prevent such promotion that can negatively affect breastfeeding practices.

**Key messages**

- Even in countries such as Cambodia, Nepal and Tanzania where point‐of‐sale promotion is restricted, promotions of BMS were observed (in nearly one‐third of stores in Phnom Penh and less than 10% in Dar es Salaam and Kathmandu).
- Limited promotion of commercially produced complementary foods was evident (less than 10% of stores had a promotion for such foods), except in Dakar Department, where promotions were found in half of stores.
- Efforts are needed to strengthen monitoring, regulation and enforcement of restrictions on the promotion of BMS.
- Manufacturers and distributors should take responsibility for compliance with national regulations and global policies pertaining to the promotion of breastmilk substitutes.

## Introduction

Promotion of breastmilk substitutes (BMS) is associated with global use of these products (Kent 2015). This promotion can be an impediment to optimal infant and young child feeding (IYCF) practices, represented by exclusive breastfeeding for the first 6 months of life followed by the timely and safe introduction of complementary foods together with continued breastfeeding until 2 years or beyond (World Health Organization (WHO) & UNICEF 2003). Breastfeeding plays a vital role in maternal and child health and nutrition and has been shown to provide long‐term benefits for a child's future educational attainment and income (Victora *et al*. 2015). Promotion of BMS, including infant formula, follow‐up formula and products for older infants commonly referred to as growing‐up milks, has been shown to negatively affect breastfeeding practices (Howard *et al*. 2000; Stewart & Guilkey 2000), and countries such as Brazil that have limited promotion of BMS have had success in improving breastfeeding practices (Cyrillo *et al*. 2009).

Despite an increased global focus on child nutrition and stunting, breastfeeding practices in Africa and Asia show room for improvement. The 2014 Demographic and Health Survey in Cambodia found that 65% of children less than 6 months of age are exclusively breastfed, a decline from the 74% in 2010 (NIS Cambodia 2011, 2015), and there is evidence that both bottle feeding (NIS Cambodia 2015) and BMS use (Prak 2014) are increasing. In Nepal, where exclusive breastfeeding through 6 months of age is generally high at 70%, early introduction of complementary foods is a concern; 3% of breastfeeding children aged 2–3 months receive some kind of solid or semi‐solid food, a figure that rises to 23% by 4–5 months of age (MOHP 2012). In Senegal, 33% of children less than 6 months of age are exclusively breastfed (ANSD 2015), and 53% of children receive a pre‐lacteal feed (provision of other liquids in the first 3 days after delivery) (ANSD 2012). Only 51% of Tanzanian infants 2–3 months of age are exclusively breastfed, and this falls to 23% by 4–5 months of age (NBS & ICF Macro 2011).

National data also indicate suboptimal complementary feeding practices in these countries, which can adversely impact child growth and development (Kothari *et al*. 2014). Only 24% of Cambodian children (NIS 2011), 24% of Nepalese children (MOHP 2012), 8% of Senegalese children (ANSD 2012) and 21% of Tanzanian children (NBS & ICF Macro 2011) aged 6–23 months meet the minimum standard with respect to all three IYCF indicators (adequate feeding frequency, minimum dietary diversity and consumption of breastmilk/other milks).

The International Code of Marketing of Breast‐milk Substitutes (the Code) and subsequent World Health Assembly resolutions prohibit point‐of‐sale promotions of BMS, specifically stating that

there should be no point of‐sale advertising, giving of samples, or any other promotion device to induce sales directly to the consumer at the retail level, such as special displays, discount coupons, premiums, special sales, loss‐leaders and tie‐in sales, for products within the scope of this Code (WHO 1981).

Early introduction of complementary foods before 6 months of age displaces breastmilk in the diet (Becker & Remmington 2014; WHO & STAG 2015) and increases likelihood of breastfeeding cessation (Qiu *et al*. 2010). Breastfeeding can be compromised when BMS products are indirectly promoted through promotion of commercially produced complementary food (CPCF) products made by the same manufacturer. This can occur when CPCF labels, promotional displays or informational materials show bottles/use of the product in a bottle, or when CPCF labels suggest use in infants under 6 months of age (either through instructions or through images of younger infants). Joint promotion of both a BMS and CPCF product together in a retail environment through displays, price discounts, informational materials or other means also simultaneously promotes these different product categories. The Draft Clarification and Guidance on Inappropriate Promotion of Foods for Infants and Young Children prepared by the WHO Scientific and Technical Advisory Group (STAG) on Inappropriate Promotion of Foods for Infants and Young Children in the fall of 2015 states that there should be ‘no cross‐promotion or brand extension to indirectly promote breastmilk substitutes via promotion of foods for infants and young children up the age of 36 months’ (WHO & STAG 2015). Specific reference is made to labelling or promotional practices such as similar colour schemes and designs, similar names and similar promotional slogans, mascots or other symbols on products manufactured by companies that also market BMS.

Various countries have adopted the Code or some provisions of the Code into national legislation and regulations. The Code has been enacted in full into national legislation in Nepal and Tanzania, and many provisions of the Code have been enacted into national law or legal action in Cambodia and Senegal (UNICEF 2011). Among the types of promotions prohibited by the Code are point‐of‐sale promotions – marketing activity taking place at commercial or retail locations – for BMS (WHO 2013). Some counties also include CPCF in national laws. Table 1 summarizes point‐of‐sale promotion restrictions according to Cambodia, Nepal, Senegal and Tanzania national legislation. Point‐of‐sale promotions for all IYCF products with an age of introduction below 12 months and below 5 years are prohibited in Nepal and Tanzania, respectively. Point‐of‐sale promotions for products with an age of introduction before 24 months are prohibited in Cambodia, unless permission has been granted from the Ministry of Health/Ministry of Information. Point‐of‐sale promotion of these products is not prohibited in Senegal, but infant formula and follow‐up formula may only be sold in pharmacies.

**Table 1:** Restrictions on BMS and CPCF product point‐of‐sale promotions in national legislation, by product category in Cambodia, Nepal, Senegal and Tanzania

Country and relevant legislation	BMS			CPCF	Product age range applicable
	Infant formula	Follow‐up formula	Growing‐up milk		
Cambodia	Promotions prohibited*	Promotions prohibited*	Promotions prohibited*	Promotions prohibited*	<24 months
Sub‐decree on marketing of products for infant and young child feeding (no. 133)‡					
Nepal	Promotions prohibited	Promotions prohibited	No prohibition on promotions	Promotions prohibited	<1 year
Mother's Milk Substitutes (Control of Sale and Distribution) Act**					
Senegal	No prohibition on promotions†	No prohibition on promotions†	No prohibition on promotions	No prohibition on promotions	<12 months
Inter‐ministerial decree establishing the conditions for marketing BMS††					
Tanzania	Promotions prohibited	Promotions prohibited	Promotions prohibited	Promotions prohibited	<5 years
National regulations for marketing of BMS and designated products‡‡					

BMS, breastmilk substitutes; CPCF, commercially produced complementary food.

* Promotions are prohibited without prior approval from the Ministry of Health/Ministry of Information.

† Sales of BMS for children less than 12 months of age are restricted to pharmacies in Senegal by Article L.511: 94–57 of the Public Health Code.

‡ Kingdom of Cambodia 2005.

** Nepal Government 1992.

†† Republic of Senegal, Ministry of Health and Social Action, Ministry of Trade 1994.

‡‡ Ministry of Health and Social Welfare, United Republic of Tanzania 2013.

In order to assess promotion levels of BMS and complementary foods, two countries in Asia and two in Africa where Helen Keller International works were selected for a study of point‐of‐sale promotion. The varying ways in which the Code has been adopted in national legislation in Cambodia, Nepal, Senegal and Tanzania may impact the messaging mothers and caregivers receive at the point of sale about IYCF. This is of particular concern given the gaps in optimal breastfeeding and complementary feeding practices observed in these four countries. In addition, although three out of four countries included in this study (Cambodia, Nepal and Tanzania) restrict point‐of‐sale promotion, ad hoc reports have illustrated that promotion often takes place despite legal restrictions (Uniting Church in Australia 2010; IBFAN 2014). However, such reports are seldom quantitative and able to assess the prevalence of such practices. Understanding the extent of promotion of commercial products is needed in order to curtail promotion that can negatively impact breastfeeding, thereby threatening infant and young child nutrition and health. The objective of this study is to assess the prevalence and characteristics of point‐of‐sale promotions of BMS and CPCF in Phnom Penh, Cambodia; Kathmandu Valley, Nepal; Dakar, Senegal; and Dar es Salaam, Tanzania.

## Methods

Point‐of‐sale promotions were assessed for two categories of commercially available IYCF products: BMS and CPCF. BMS are defined in this study to include infant formula (recommended from birth up to 6 months of age), follow‐up formula (recommended from 6–11 months of age) and growing‐up milks usually marketed for children 12+ months of age (Przyrembel & Agostoni 2013; Vandenplas *et al*. 2014; Piwoz & Huffman 2015). CPCF includes any commercially produced food or beverage product, excluding BMS, which displays a label indicating that the product is intended for children younger than 2 years of age. In order to record all IYCF brands and products available for sale in the country, a comprehensive product scoping list of available BMS and CPCF in Phnom Penh, Cambodia; Kathmandu Valley, Nepal; Dakar Department, Senegal; and Dar es Salaam, Tanzania, was developed through visits to local stores, Internet research, interviews with local nutrition experts, contacting local offices of BMS and CPCF manufacturers/distributors and, where available, obtaining official lists of registered products. Products were identified as different based on the following variables: brand name, sub‐brand name, descriptive name, age category and serving size (whether it was a single, double or multiple serving). One flavour and size variant of each product was included in the list. This product scoping list was used to guide data collection.

Stores were included in this study if they sold at least one BMS product or at least one CPCF. A purposive sample of approximately 10 large stores, which were chosen for their larger volume and range of products (including national chain grocery stores, supermarkets, baby stores and chain pharmacies known to sell either BMS or CPCF) and a random sample of approximately 20 smaller stores with a smaller selection of products (including corner/convenience stores, neighbourhood stores/kiosks and independent pharmacies) selling BMS or CPCF were made in each city (Table 2). Purposive sampling of large stores took place because it was expected that they would carry the majority of the IYCF products available for sale nationally based on research conducted by Sweet *et al*. (2012) and were strategically selected based on Helen Keller International in‐country staff knowledge that they potentially stocked the greatest variety of CPCF products. Additional detail on store and product sampling procedures can be found in Appendix 1 of Sweet *et al*. 2016.

**Table 2:** Number of stores visited and number of administrative units included in store sampling in each city

	Total number of stores sampled	Store characteristics		Administrative units sampled for random selection of small stores
		No. of large stores	No. of small stores	
Phnom Penh	29	11	18	8 (19.5%) of the 41 *sangkats* making up the four urban khans
Kathmandu Valley	31	9	22	11 (19.3%) of the 57 wards making up the two urban municipalities
Dakar Department	31	9	22	9 (50%) of the 18 urban borough communes making up four urban boroughs
Dar es Salaam	30	10	20	10 (27%) of the 37 urban wards making up three municipal councils

In addition, a random sample of 20–50% of administrative units in each city was chosen for inclusion so that approximately 10 units were sampled in each site. This was carried out by listing and assigning numbers to all administrative units in the city and selecting units for inclusion using an online random number generator (https://www.randomizer.org/) after determining the sampling interval needed in each city to include the intended sample of 10 administrative units. The variation in per cent of geographic areas (represented by administrative units) covered in each city reflects an attempt to randomly sample a comparable number of stores in each city from similarly sized samples of administrative units. Within each administrative unit, approximately two small stores were sampled using a random‐start convenience sampling strategy using the administrative office of each selected unit as a starting point. In Phnom Penh and Dakar, one corner/convenience store and one small grocery/neighbourhood store per administrative unit and one small pharmacy in every two small administrative units were located. In Kathmandu Valley and Dar es Salaam, one to two corner/convenience stores in each administrative unit and one small pharmacy in every two administrative units were included in the sample. This mixed approach is a compromise between the time‐intensive process of compiling a comprehensive list of stores selling specified products in order to randomly sample stores throughout the city and selecting a purposive selection of stores based on local expert opinion of stores likely to sell these products.

If a sampled small store did not sell at least one IYCF product (either a BMS or a CPCF product) when visited, another small store nearest to the sampled store was then visited; if it sold BMS or CPCF, it was included in the study to replace the initially sampled store. Twelve small stores in Dar es Salaam, seven in both Phnom Penh and Dakar and two in Nepal were eliminated from the initial sample because they sold neither product, BMS or CPCF. The final sample included 29–31 stores in each site (Table 2), for a total of 121 stores. Grocery stores and supermarkets were common in all sites; however, baby stores that sold BMS/CPCF were only common in Phnom Penh (Table 3). Pharmacies made up 22.6% of Dakar stores included in this study.

**Table 3:** Number and per cent of stores included by type in each city

Store type	Phnom Penh	Kathmandu Valley	Dakar Department	Dar es Salaam	Total
Large stores (purposively sampled)					
Grocery chain stores	5	4	2	4	17
Independent grocery stores/supermarkets	1	5	2	4	12
Baby stores	4	—	—	—	4
Chain pharmacies	1	—	3	2	6
Sub‐total of large stores	11	9	9	10	39
Small stores (randomly sampled)					
Corner/convenience stores	7	17	9	16	49
Small grocery/neighbourhood stores	7		9		16
Small pharmacies	4	5	4	4	17
Sub‐total of small stores	18	22	22	20	82
Total for all stores	29	31	31	30	121

Point‐of‐sale promotions assessed in this study include price‐related promotions (coupons/stamps, discounts and special discount sales), displays (brand shelves, special shop windows detached from a shelf, placards/posters/banners, shelf tags/talkers/wobblers/screamers and new product launches), information materials (leaflets/pamphlets/flyers), free gifts (to customers), product samples and company representatives in stores.

Data collection was conducted in each study site by a team of two to three investigators, who had received a 3‐day training on standardized methods to be used at all four sites from the study coordinators prior to data collection. Data collection was carried out from June to August 2013.

The list of scoped products in each country was used during store visits to record the number of unique products available for sale. Any point‐of‐sale promotions observed in the stores were recorded and photographs taken, when permitted by store owners (photos were not allowed in one store in Phnom Penh and one store in Kathmandu Valley). Both data collection forms and photographs were uploaded to an online cloud‐based storage system and reviewed by study coordinators to ensure completeness and data quality. Any inconsistencies were discussed with the data collectors and rectified.

A point‐of‐sale promotion was defined as an individual occurrence of promotional activity for one or more BMS or CPCF products at a retail location, such as a single display, a price‐related promotion or an informational brochure promoting one or several products. The proportion of stores with promotions was based on all sampled stores selling IYCF products in each site. An ‘individual product promotion’ was counted each time a different product appeared in a point‐of‐sale promotion. Accordingly, a display containing or depicting six unique BMS products would be recorded as one point‐of‐sale promotion and six individual product promotions. Each different product type was counted only once in each promotion even if multiple units of that product were represented in the promotion (i.e. a shelf display with multiple packages of the same infant formula). If a promotion featured both BMS and CPCF products, it was counted as two separate promotions, one for each category, and then later described as a single example of joint promotion between BMS and CPCF products. The percentage of products promoted was calculated by dividing the number of different products appearing in individual product promotions by the number of different IYCF products available in stores.

In order to determine if point‐of‐sale promotions originated from the manufacturer/distributor or the retailer, photos of each promotion were assessed by observing the materials used for the promotion (e.g. professional printed materials vs. basic computer‐printed/handwritten materials), assessing the language and terminology used in the promotion (scientific information regarding the product vs. basic description), assessing details on the promotion that clearly linked it to either the manufacturer/distributor or retailer (contact information, promotional dates set by retailer, etc.) and assessing if the same promotion existed at multiple distinct stores. In some cases, the origin of the point‐of‐sale promotion was unclear and was then recorded as such.

Data were entered into Microsoft Excel 2010 and analysed in spss version 21 (IBM, Armonk, NY, USA) to determine the frequency of product availability by brand, manufacturer, category, and frequency and type of promotions. Point‐of‐sale promotions are presented as the proportion of the stores promoting at least one product and also as the number of promotions and individual promotions in each site.

## Results

### Types of BMS and CPCF products sold in retail locations

Although stores were selected for inclusion in the study based on selling at least one BMS or CPCF product, there was variation in the type of BMS and CPCF products available across the urban areas sampled. BMS products were sold in nearly all (over 90%) of the sampled stores in Phnom Penh, Kathmandu and Dar es Salaam. BMS were sold in only two‐thirds (67.7%) of stores in Dakar Department. Infant and follow‐up formula can only legally be sold in pharmacies in Dakar Department; all seven pharmacies in Dakar Department sold at least one infant formula, follow‐up formula and growing‐up milk. Growing‐up milks were sold in less than a third of stores in Dar es Salaam (30.0%), almost two‐thirds of stores in Phnom Penh (62.1%) and nearly all sampled stores in Kathmandu Valley (93.5%). In Dakar Department, they were sold in all (100.0%) of the seven pharmacies and 58.3% of the other 24 stores (67.7% of total stores).

In all cities, infant cereals were the most commonly available CPCF in stores that sold IYCF products; 62.1%, 100.0%, 100.0% and 73.3% of stores sold at least one infant cereal product in Phnom Penh, Kathmandu Valley, Dakar and Dar es Salaam, respectively. In Phnom Penh, infant snacks (including crackers, biscuits, rusks, yogurt covered snacks, dried fruit, rice cakes and grain puffs), and in Dakar Department, purees and juices/teas/water were also prevalent, with more than 50% of stores selling at least one of these products.

Among stores that sold IYCF products, the greatest number of different BMS products was available for sale in Phnom Penh (Table 4). The greatest diversity of manufacturers of BMS was also found in Phnom Penh; at least 25% of stores stocked products from the following BMS manufacturers: Abbott, Dumex, France Bebe, Friesland Campina, Laboratories Gilbert, Lypack, Namyang, Nestlé and Wyeth. 1Wyeth was acquired by Pfizer in 2009 (Thomson Financial 2009). In 2012, Nestlé bought Pfizer's infant nutrition division (Pfizer Nutrition) including Wyeth and associated brands (e.g. S‐26, S‐26 Gold, SMA and Promil) (Press Release, Nestle 2012). In this study, the manufacturer and brand names captured and reported were those provided on the product label. In total in Phnom Penh, 26 different manufacturers of BMS were found in stores. In Kathmandu Valley, only three manufacturers' products were found, with Nestlé products nearly ubiquitous across stores; 28 (96.8%) stores sold at least one BMS product manufactured by Nestlé, while other manufacturers' products were available in less than 10% of stores. Seven BMS manufacturers were found in Dakar Department and six in Dar es Salaam. Danone and Nestlé products were found in at least 25% of stores in Dakar Department, while Aspen Nutritionals and Nestlé products were sold in at least 25% of stores in Dar es Salaam. No local manufacturers of BMS products were found in any city.

**Table 4:** Number/per cent of different BMS, by type, sold in at least one store

	Phnom Penh		Kathmandu Valley		Dakar Department		Dar es Salaam	
	*n* (%)		*n* (%)		*n* (%)		*n* (%)	
BMS products	112		14		36		17
Infant formula	47	42.0%	5	37.5%	16	44.4%	9	52.9%
Follow‐up formula	34	30.4%	5	37.5%	15	44.0%	5	29.4%
Growing‐up milk	31	27.7%	4	28.6%	5	13.9%	3	17.6%

BMS, breastmilk substitutes.

There were between 22 and 84 different CPCFs sold in sampled stores in the four cities, with the greatest number of different individual CPCF products found in Dakar. The majority of products were imported. In Phnom Penh, only 1.4% of these were produced by a local (national) manufacturer, and in Kathmandu, Dakar Department and Dar es Salaam, respectively, 36.4%, 4.8% and 30.8% of CPCF products came from local manufacturers (Table 5).

**Table 5:** Number/per cent of different CPCFs, by type, sold in at least one store

	Phnom Penh		Kathmandu Valley		Dakar Department		Dar es Salaam	
	*n* (%)		*n* (%)		*n* (%)		*n* (%)	
CPCF products	69		22		84		25
Cereals	17	24.6%	21	95.5%	36	42.9%	19	76.0%
Purees	29	42.0%	1	4.5%	39	46.4%	4	16.0%
Snack/finger foods	15	21.7%	0	0.0%	3	3.6%	2	8.0%
Juices/waters/teas	8	11.6%	0	0.0%	6	7.1%	0	0.0%

CPCF, commercially produced complementary food.

### Point‐of‐sale promotions for breastmilk substitutes

Across the four sites, BMS point‐of‐sale promotions were found in 3.2–37.9% of stores. The most stores with promotions were found in Phnom Penh, and the fewest were found in Kathmandu Valley (Table 6). In Phnom Penh and Dakar, where point‐of‐sale promotions for BMS were found in over one‐third of stores, the majority of promotions were determined to be manufacturer initiated. Promotions found in these two cities tended to include multiple products; 71 and 94 individual product promotions were found, respectively. These individual product promotions included just less than a third of all products available in sampled stores in Phnom Penh, while almost all of the different BMS products available for sale in Dakar were represented. In Dakar, where infant formula and follow‐up formula can only be sold in pharmacies, promotions for these categories of products were only seen in pharmacies. Sixty‐five per cent (13/20) of all BMS promotions in Dakar took place in pharmacies. Fewer promotions were seen in Kathmandu Valley and Dar es Salaam, although at least one product from each category of BMS (infant formula, follow‐up formula and growing‐up milk) was found in a promotion. Under a third of products available in stores in Kathmandu Valley and Dar es Salaam were found in any promotion, with much fewer individual product promotions in each promotion. The singular promotion found in Kathmandu Valley was determined to be manufacturer/distributor initiated. This promotion also featured CPCF products, discussed in the following section. Both promotions for BMS in Dar es Salaam originated with the retailer.

**Table 6:** Number and proportion of all stores selling IYCF products with at least one POS promotion for BMS, number of POS promotions and number of individual product promotions

	Phnom Penh		Kathmandu Valley		Dakar Department		Dar es Salaam	
	*n*/no. of stores		*n*/no. of stores		*n*/no. of stores		*n*/no. of stores	
Stores with at least one POS promotion for BMS	11/29		1/31		11/31		2/30
Infant formula	9/29		1/31		5/31		2/30	
Follow‐up formula	9/29		1/31		5/31		1/30	
Growing‐up milks	9/29		1/31		11/31		1/30	
Percentage of all products found in stores that were promoted	30.4		28.6		83.3		35.3	
Number of POS promotions	30		1		20		2	
Number (%) of manufacturer‐driven POS promotions for BMS	18	60.0%	1	100.0%	19	95.0%	0	0.0%
Number (%) of manufacturer‐driven POS promotions for BMS by manufacturer (for manufacturers with at least 10% of all promotions)								
	Phnom Penh		Kathmandu Valley		Dakar Department		Dar es Salaam	
	*n*	(%)	*n*	(%)	*n*	(%)	*n*	(%)
Abbott	4	22.2						
Danone					8	42.1		
Gilbert Laboratories	5	27.8						
Nestlé			1	100.0	9	47.3		
Wyeth	4	22.2						
Other	5	27.8			2	10.5		
Number of total individual product promotions for BMS	71		4		94		6	

BMS, breastmilk substitutes; IYCF, infant and young child feeding; POS, point of sale.

Manufacturer listed on product label.

In all countries, the most common type of BMS promotion observed was promotional product displays (e.g. posters, banners, branded racks and special displays in a window). Just over half (56.7%) of BMS point‐of‐sale promotions in Phnom Penh and almost all (95% or 19/20) of promotions in Dakar were product displays. The singular promotion in Kathmandu Valley and one of the two promotions in Dar es Salaam were displays. Other categories of promotions observed for BMS included informational materials, free gifts and price‐related promotions.

### Point‐of‐sale promotions of commercially produced complementary foods

In Phnom Penh and Kathmandu Valley, point‐of‐sale promotions for CPCF products were less prevalent than those for BMS. In Dakar, CPCF promotions were more prevalent than BMS, although they were promoted in only five out of seven pharmacies. BMS and CPCF products were found in promotions in the same number of stores in Dar es Salaam (Table 7). There were fewer than 10 total point‐of‐sale promotions for CPCF in Phnom Penh, Kathmandu Valley and Dar es Salaam. There were substantially more CPCF promotions in Dakar, at 33 promotions and 155 individual product promotions. It was determined that the majority of CPCF promotions in Dakar were manufacturer driven, while the proportion was lower for the other countries, and similarly to BMS promotions, all were retailer driven in Dar es Salaam. These promotions featured 7.2–66.7% of products available for sale in each country.

**Table 7:** Number and proportion of all stores selling IYCF products with CPCF promotions, number of POS promotions and number of individual product promotions

	Phnom Penh		Kathmandu Valley		Dakar Department		Dar es Salaam	
	*n*/no. of stores		*n*/no. of stores		*n*/no. of stores		*n*/no. of stores	
Stores with at least one POS promotion for CPCF	6/29		2/31		15/31		2/30	
Infant cereal	2/29		2/31		14/31		2/30	
Purees	0/29		0/31		7/31		0/30	
Snacks	2/29		0/31		0/31		0/30	
Juice/water	3/29		0/31		3/31		0/30	
Percentage of all products found in stores that were promoted	7.2		22.7		66.7		16.0	
Number of POS promotions	7		2		33		3	
Number (%) of manufacturer‐driven POS promotions for CPCF	2	28.6%	1	50.0%	27	81.8%	0	0.0%
Number (%) of manufacturer‐driven POS promotions for CPCF by manufacturer (for manufacturers with at least 10% of all promotions)								
	Phnom Penh		Kathmandu Valley		Dakar Department		Dar es Salaam	
	*n*	(%)	*n*	(%)	*n*	(%)	*n*	(%)
Celia/Lactalis int'l	1	50.0						
Danone					7	25.9		
Nestlé			1	100.0	13	48.1		
PPM	1	50.0						
Other					7	25.9		
Number of total individual product promotions for any CPCF	7		5		155		5

BMS, breastmilk substitutes; CPCF, commercially produced complementary food; IYCF, infant and young child feeding; POS, point of sale.

Manufacturer listed on product label.

Similar to promotions for BMS, most promotions for CPCF were product displays: 90.9% (30/33) of promotions in Dakar and one out of one promotion in Kathmandu Valley. However, in Dar es Salaam, only one out of three total promotions was a display, while the other two were price‐related promotions. In Phnom Penh, price‐related promotions for CPCF were the most prevalent; 71.4% of products were price‐related promotions.

Placement of BMS and CPCF products together in the same point‐of‐sale promotion was observed in stores in three of the four study sites. Joint promotions featuring at least one BMS and one CPCF together in the same promotion were found in five out of seven pharmacies and 20.8% (*n* = 5) of other stores in Dakar Department. All joint promotions in pharmacies featured BMS and CPCF products made by a single manufacturer; one of the five other stores had one retailer‐driven joint promotion featuring multiple (five) manufacturers' products.

Both stores where promotions were found in Dar es Salaam had one promotion where BMS and CPCF were promoted together. One of these stores had a retailer‐driven promotion that featured CPCF and BMS products produced by a single manufacturer, and one store had a retailer‐driven promotion that included multiple manufacturers' products.

No instances of joint promotion featuring a BMS and CPCF together were found in any stores in Phnom Penh.

## Discussion

The results of this study indicate that commercial IYCF products are commonly available in stores in Phnom Penh, Kathmandu Valley, Dakar Department and Dar es Salaam and that point‐of‐sale promotion of these products can be found in all four cities.

A link between availability of a variety of different products in stores and use of these products among consumers is not immediately clear from the results of this research. However, recent studies may provide some insight into possible correlations. A recent survey of urban mothers in Phnom Penh found that rates of use of BMS are higher than other commercial IYCF products. For children 6–23 months of age, 29.3% of children had consumed a BMS in the previous day, while only 5.4% consumed a CPCF (Pries *et al*. 2016a). The proliferation of BMS products available for sale (more than three times the number of different products sold and manufacturers represented than the other three countries) is happening in parallel to declining exclusive breastfeeding rates in Cambodia (NIS Cambodia 2011; Prak *et al*. 2014; NIS Cambodia 2015). In Dakar, a similar survey found a different situation, with 20.2% of children 6–23 months of age having consumed BMS while 26.1% consumed CPCF (Feeley *et al*. 2016). A greater diversity of CPCF products was found in Dakar than the other three cities, while fewer different BMS manufacturers and products were found for sale (a distribution pattern that may be affected by the restriction of these products to sale in pharmacies). In Kathmandu, a relatively small selection of BMS products was available in nearly all sampled stores. Nepal DHS data indicate high rates of exclusive breastfeeding (MOHP 2012), and recent evidence suggests low use of BMS products; a cross‐sectional survey found that only 7.5% of children 6–23 months of age consumed BMS (Pries *et al*. 2016b). This same study indicated that 24.6% of these children had consumed a CPCF (Pries *et al*. 2016b). In Dar es Salaam, where a limited variety of both types of products were found, utilization of both BMS and CPCF is reported to be low, with only 4.8% of children 6–23 months of age consuming BMS and 3.1% consuming CPCF (Vitta *et al*. in press). This suggests that use of commercial IYCF products is likely not influencing the low rates of exclusive breastfeeding and low achievement of appropriate dietary diversity in complementary foods (NBS & ICF Macro 2011) among children under 2 in Tanzania; these dietary gaps are perhaps the result of other factors.

The strength of national legislation restricting the point‐of‐sale promotion of commercial IYCF products seems to affect the prevalence of point‐of‐sale promotions. Of the four countries included in this study, Tanzania and Nepal have the most restrictive legislation; point‐of‐sale promotions are restricted for all products with an age of introduction less than 5 years and up to 12 months in each country, respectively. Less than 10% of sampled stores in these cities had promotions for BMS or CPCF. Higher levels of point‐of‐sale promotion were seen in Phnom Penh and Dakar, where legislation is less restrictive on point‐of‐sale promotions. Cambodia restricts point‐of‐sale promotions of BMS and CPCF up to the age of 24 months, but permission to do so can be granted by the Ministry of Health/Ministry of Information. No promotions in this study displayed a license of approval, and there is no stipulation in national policy regarding whether this permission must be displayed on approved promotions, so it is unclear if promotions observed were legal or illegal. Senegal permits point‐of‐sale promotions of all IYCF products unless they are sold within the health system. The high degree of point‐of‐sale promotion of products in these two sites is concerning. Over a third of stores in each city had point‐of‐sale promotions for BMS. Infant formula and follow‐up formula promotions were less prevalent in Dakar because they only occurred in pharmacies, which represented 22.6% (7/31) of the total sample of stores. Point‐of‐sale promotions for CPCF took place in about one‐third and one half of stores in Phnom Penh and Dakar, respectively.

Growing‐up milks, which in this study were defined based on previously published sources as fortified milk products for children 12+ months of age, were observed in promotions in all four sites. This occurred legally in some instances and illegally in others. Senegal does not restrict point‐of‐sale promotion of these products, and if the age of introduction is greater than 12 months, they are not restricted in Nepal. Cambodia restricts promotions for products with an age of introduction up to 24 months without permission, and Tanzania restricts promotions for all foods for children under 5 years of age. Point‐of‐sale promotions observed for growing‐up milks represent a means for manufacturers to advertise these products that often have similar names to their infant formula or form part of the same product line. Branding and the use of numbered stages for these products (e.g. naming the infant formula ‘Step 1’ and the growing up milk ‘Step 3’) can lead to confusion and result in feeding young children a food that is inappropriately formulated for their age (Pereira *et al*. in press). Restriction of the promotion of growing‐up milks with an age of introduction beyond 12 months is particularly important because mothers/caregivers often cannot differentiate between different categories of BMS being promoted and may interpret them to be part of a general category of ‘formula’ suitable for all infants and young children (Berry *et al*. 2010; Cattaneo *et al*. 2015). In order to avoid this confusion among caregivers, Cambodia and Tanzania's laws with regard to growing‐up milks must be enforced, while Nepal and Senegal's laws must be strengthened to cover these products. Joint promotion of BMS and CPCF products together in point‐of‐sale promotions occurred in Kathmandu Valley, Dakar Department and Dar es Salaam stores. Direct simultaneous point‐of‐sale promotion of these types of products together can similarly lead to the danger of early introduction of complementary foods or confusion among age and product categories. Such joint promotional activities should also be restricted in order to protect exclusive breastfeeding until 6 months followed by adequate and timely introduction of healthy complementary foods.

In order to support optimal infant feeding practices, enforcement of legislation restricting promotions of BMS products must be strengthened. In Cambodia, clarity is required on the process by which manufacturers must obtain approval for point‐of‐sale promotions and under what conditions such approval is given. Senegal's regulations must also be expanded to prohibit promotion of BMS products at the point of sale. The current legislation restricting BMS promotion in Nepal and Tanzania must also be adequately enforced. In addition, manufacturers must be responsible for compliance with the Code and national legislation where their products are sold; all BMS and most CPCF products observed in this study were manufactured by international companies. With the exception of Dar es Salaam, where no manufacturer‐driven promotions were found, 60.0–100.0% of point‐of‐sale promotions for BMS and 40.0–81.8% of point‐of‐sale promotion for CPCF in the other sites were judged to have been initiated by manufacturers/distributors rather than the retailers at the point of sale. A recent study has noted that all participants along the supply chain need to be engaged to promote and protect breastfeeding (Barennes *et al*. 2015). The results of this study also indicate that manufacturers, distributors, wholesalers/importers and retailers should take responsibility for compliance with national regulations and global guidance on point‐of‐sale promotion of BMS.

Limitations of this study include the difficulty in developing a standardized protocol for sampling administrative units in each city to achieve a representative geographic sample of possible retail locations to visit. The high degree of variability in type and distribution of stores across each metropolitan area also posed a problem for sampling. The decision to sample only stores that sold IYCF products may be considered a limitation in terms of accurately reflecting the availability of these products in retail locations across each city; if a wider sample of retail stores had been included, there would have been a lower proportion of stores with promotions. However, it seemed reasonable to include only stores selling products because inclusion of any store could have limited the ability to assess promotions where they were likely to occur.

Creating a random sample of these stores proved to be problematic. Given the lack of comprehensive lists or rosters of retail areas, and the degree of variability among types of stores found in each site, a combination of purposive sampling of large stores and random‐start convenience sampling of smaller stores allowed for a diverse sample of retail environments (in terms of geographic area and store characteristics). This is likely not representative of all stores selling IYCF products in each city. Variability in products expected to be sold in retail locations and the actual prevalence of products were observed. The complexity of the IYCF market made it difficult to assess the origin and ultimate manufacturer of some products; thus, only information on the product label was reported. The origins of promotions were determined based on the characteristics of the promotions and could not be confirmed conclusively. It should be noted that in addition to the observation of practices at physical points of sale assessed in this study, caregivers can be exposed to promotions for commercial products through other retail avenues including online sales.

## Conclusions

Point‐of‐sale promotions of BMS are counter to the WHO International Code of Marketing of Breast‐milk Substitutes and in national legislation in many countries, including Cambodia, Nepal and Tanzania, with their sales restricted to pharmacies in Senegal. Despite this, such promotions were observed in the four countries included in this study. Voluntary compliance with the WHO Code and national laws restricting point‐of‐sale promotion of IYCF products is not occurring in retail stores in Phnom Penh, Kathmandu Valley, Dakar Department or Dar es Salaam. This research highlights the need for both the strengthening of national legislation itself and improvement in monitoring and compliance on the part of manufacturers, distributors and retailers who conduct point‐of‐sales promotions.

## Source of funding

Funding for this research was provided by the Bill & Melinda Gates Foundation.

## Conflicts of interest

The authors have no conflicts of interest to declare.

## Contributions

MC analyzed the data and prepared the manuscript. LS, CP, SH and EZ conceptualized and designed the study. AC, NS, IA, SD, CM and KM collected the data, which was overseen by CP and LS. SH guided data analysis and provided technical insight. All authors reviewed and provided input on the final article.

## References

[mcn12272-bib-0001] Agence Nationale de la Statistique et de la Démographie (ANSD) [Sénégal], et ICF International (2012) Enquête Démographique et de Santé à Indicateurs Multiples au Sénégal (EDS‐MICS) 2010–2011. ANSD et ICF International: Calverton, Maryland, USA.

[mcn12272-bib-0002] Agence Nationale de la Statistique et de la Démographie (ANSD) [Sénégal], et ICF International (2015) Sénégal: *Enquête Démographique et de Santé Continue* (*EDS‐Continue 2014*). ANSD et ICF International: Rockville, Maryland, USA.

[mcn12272-bib-0003] Barennes H. , Slesak G. , Goyet S. , Aaron P. & Srour L.M. (2015) Enforcing the International Code of Marketing of Breast‐milk Substitutes for better promotion of exclusive breastfeeding: can lessons be learned? Journal of Human Lactation, 1–8. Available at: http://jhl.sagepub.com.oca.ucsc.edu/content/early/2015/09/26/0890334415607816 (Accessed 28 September 2015). 10.1177/0890334415607816 26416439

[mcn12272-bib-0004] Becker G. & Remmington T. (2014) Early additional food and fluids for healthy breastfed full‐term infants. Cochrane Database of Systematic Reviews 11, CD006462. DOI: 10.1002/14651858.CD006462.pub3. 25420475

[mcn12272-bib-0005] Berry N.J. , Jones S. & Iverson D. (2010) It's all formula to me: women's understandings of toddler milk ads. Breastfeeding Review 18, 21–30. 20443436

[mcn12272-bib-0006] Cattaneo A. , Pani P. , Guidetti M. , Mutti V. , Guidetti C. , Knowles A. *et al.* (2015) Advertisements of follow‐on formula and their perception by pregnant women and mothers in Italy. Archives of Disease in Childhood 100, 323–328 Published Online First: 15 December 2014. 10.1136/archdischild-2014-306996 25512963

[mcn12272-bib-0007] Cyrillo D.C. , Sarti F.M. , Mercier E. , Farina Q. & Mazzon J.A. (2009) Two decades of the Brazilian Standard for Marketing of Baby Food: are there reasons to celebrate? Pan American Journal of Public Health. DOI: 10.1590/S1020-49892009000200006. 19531308

[mcn12272-bib-0008] Feeley A.B. , Ndeye Coly A. , Sy Gueye N.Y. , Diop E.I. , Pries A. , Champeny M. , Zehner E. , Huffman S.L. (2016) Promotion and consumption of commercially produced foods among children: situation analysis in an urban setting in Senegal. Maternal & Child Nutrition 12 (Suppl. 2), 64–76. 10.1111/mcn.12304 PMC5071683 27061957

[mcn12272-bib-0010] Howard C. , Howard F. , Lawrence R. , Andresen E. , DeBlieck E. & Weitzman M. (2000) Office prenatal formula advertising and its effect on breast‐feeding patterns. Obstetrics & Gynecology 95, 296–303. 10674597 10.1016/s0029-7844(99)00555-4

[mcn12272-bib-0012] International Baby Food Action Network (IBFAN) (2014) Breaking the Rules Stretching the Rules 2014. IBFAN Sdn Bhd: Penang, Malaysia Available at: http://www.ibfan-icdc.org/files/1__Preliminary_pages_5-2-2014.pdf (Accessed 19 June 2015).

[mcn12272-bib-0013] Kent G. (2015) Global regulation of infant formula. International Breastfeeding Journal 10, 6 DOI: 10.1186/s13006-014-0020-7. 25784954 PMC4362817

[mcn12272-bib-0014] Kingdom of Cambodia (2005) Sub‐decree on the marketing of products for infant and young child feeding, No.133.

[mcn12272-bib-0015] Kothari M.T. , Abderrahim N. , Coile A. & Cheng Y. (2014) Nutritional Status of Women and Children. ICF International: Rockville, Maryland, USA.

[mcn12272-bib-0016] Ministry of Health and Population (MOHP) Nepal, New ERA & ICF International Inc . (2012) Nepal Demographic and Health Survey 2011. Nepal Demographic and Health Survey 2011: Ministry of Health and Population, New ERA, and ICF International.

[mcn12272-bib-0017] Ministry of Health And Social Welfare, United Republic of Tanzania (2013) The Tanzania Food, Drugs and Cosmetics Act, marketing of foods and designated products for infants and young children regulations.

[mcn12272-bib-0018] National Bureau of Statistics [Tanzania] & ICF Macro (2011) Tanzania Demographic and Health Survey 2010. National Bureau of Statistics & ICF Macro: Dar es Salaam, Tanzania.

[mcn12272-bib-0019] National Institute of Statistics (NIS) [Cambodia], Directorate General for Health and ICF International (2015) Cambodia Demographic and Health Survey 2014. National Institute of Statistics, Directorate General for Health, and ICF International: Phnom Penh, Cambodia and Rockville, Maryland, USA.

[mcn12272-bib-0020] National Institute of Statistics (NIS) [Cambodia], Directorate General for Health and ICF Macro (2011) Cambodia Demographic and Health Survey 2010. Phnom Penh, Cambodia and Calverton, Maryland, USA: National Institute of Statistics, Directorate General for Health, and ICF Macro.

[mcn12272-bib-0021] Nepal Government (then His Majesty's Government) (1992) The Mother's Milk Substitutes (Control of Sale and Distribution) Act, 2049 (1992) Act No. 39 of 2049 (1992 A.D.). Kathmandu, Volume 42. 29th Mangshir 2049 (Supplementary 48).

[mcn12272-bib-0022] Nestlé (2012) Nestlé to acquire Pfizer Nutrition in strategic move to enhance its position in global infant nutrition. Press Release. Vevey, Switzerland.

[mcn12272-bib-0023] Pereira C. , Ford R. , Feeley A. , Badham J. , Mengkheang K. , Adhikary I. *et al.* (2016) Assessment of the labels of commercially produced complementary foods sold in Cambodia, Nepal, Senegal and Tanzania. Maternal & Child Nutrition 12 (Suppl. 2), 106–125. 10.1111/mcn.12268 PMC5071699 27061960

[mcn12272-bib-0024] Piwoz E.G. & Huffman S.L. (2015) The impact of marketing of breast‐milk substitutes on WHO‐recommended breastfeeding practices. Food and Nutrition Bulletin, 36(4), 373–386. DOI: 10.1177/0379572115602174. 26314734

[mcn12272-bib-0025] Prak S. , Dahl M.I. , Oeurn S. , Conkle J. , Wise A. & Laillou A. (2014) Breastfeeding trends in Cambodia, and the increased use of breast‐milk substitute – why is it a danger? Nutrients 6, 2920–2930. 25054552 10.3390/nu6072920 PMC4113769

[mcn12272-bib-0026] Pries A. , Huffman S.L. , Adhikary I. , Upreti S.R. , Dhungel S. , Champeny M. *et al.* (2016a) High consumption of commercial food products among children less than 24 months of age and product promotion in Kathmandu Valley, Nepal. Maternal & Child Nutrition 12 (Suppl. 2), 22–37. 27061954 10.1111/mcn.12267 PMC5071716

[mcn12272-bib-0027] Pries A. , Huffman S.L. , Mengkheang K. , Kroeun H. , Roberts M. , Champeny M. *et al.* (2016b) Common utilization of commercial food products during the complementary feeding period and reported commercial promotions for these products among Phnom Penh mothers of children 6–23 months of age. Maternal & Child Nutrition 12 (Suppl. 2), 38–51.27061955

[mcn12272-bib-0028] Przyrembel H. & Agostoni C. (2013) Growing‐up milk: a necessity or marketing? World Review of Nutrition and Dietetics 108, 49–55. 24029786 10.1159/000351484

[mcn12272-bib-0029] Qiu L. , Binns C. , Zhao Y. , Lee A. & Xie X. (2010) Breastfeeding practice in Zhejiang Province, PR China, in the context of melamine‐contaminated formula milk. Journal of Health, Population and Nutrition 28, 189–198. 10.3329/jhpn.v28i2.4891 PMC2980882 20411683

[mcn12272-bib-0030] Republic of Senegal; Ministry of Health and Social Action, Ministry of Trade and Handicrafts (1994) *005969. Inter‐ministerial decree establishing the conditions for marketing breast‐milk substitutes.

[mcn12272-bib-0032] Stewart J.F. & Guilkey D.K. (2000) Estimating the health impact of industry infant food marketing practices in the Philippines. The Journal of Development Studies 36, 50–77.

[mcn12272-bib-0033] Sweet L. , Jerling J. & Van Graan A. (2012) Field‐testing of guidance on the appropriate labelling of processed complementary foods for infants and young children in South Africa. Maternal & Child Nutrition 9, 12–34. 10.1111/mcn.12019 PMC6860532 23167582

[mcn12272-bib-0044] Sweet L. , Pereira C. , Ford R. , Feeley A.B. , Badham J. , Mengkheang K. *et al* (2016). Assessment of corporate compliance with guidance and regulations on labels of commercially produced complementary foods sold in Cambodia, Nepal, Senegal and Tanzania. Maternal & Child Nutrition 12 (Suppl. 2), 106–125. 10.1111/mcn.12268 PMC5071699 27061960

[mcn12272-bib-0034] Thomson Financial (2009) PFE – Pfizer to acquire Wyeth, creating the world's premier biopharmaceutical company. Available at: http://www.pfizer.com/files/investors/presentations/q4_transcript_012609.pdf (Accessed 13 August 2015).

[mcn12272-bib-0035] UNICEF (2011) National implementation of the International Code of Marketing of Breastmilk Substitutes (April 2011). Nutrition Section, UNICEF, New York.

[mcn12272-bib-0036] Uniting Church in Australia (2010) Unethical marketing of infant formula and breastmilk substitutes in Cambodia 2009. Justice and International Mission Unit Synod of Victoria and Tasmania Uniting Church in Australia: Melbourne, Victoria Australia Available at: http://camnut.weebly.com/uploads/2/0/3/8/20389289/2010marketingofinfantformulareporten.pdf (Accessed 13 August 2015).

[mcn12272-bib-0037] Vandenplas Y. , De Ronne N. , Van De Sompel A. , Huysentruyt K. , Robert M. , Rigo J. *et al.* (2014) A Belgian consensus‐statement on growing‐up milks for children 12–36 months old. European Journal of Pediatrics 173, 1365–1371. 24764116 10.1007/s00431-014-2321-7

[mcn12272-bib-0038] Victora C.G. , Horta B.L. , de Mola C.L. , Quevedo L. , Pinheiro R.T. , Gigante D.P. *et al.* (2015) Association between breastfeeding and intelligence, educational attainment, and income at 30 years of age: a prospective birth cohort study from Brazil. The Lancet Global Health 3, 199–205. 10.1016/S2214-109X(15)70002-1 PMC4365917 25794674

[mcn12272-bib-0039] Vitta B. , Benjamin M. , Pries M. , Champeny M. , Huffman S.L. & Zehner E. (2016) Health systems study findings from Dar es Salaam, Tanzania. Maternal & Child Nutrition 12 (Suppl. 2), 77–90. 27061958 10.1111/mcn.12292 PMC5071773

[mcn12272-bib-0040] World Health Organization (WHO) (1981) International Code of Marketing of Breast‐milk Substitutes. Geneva.

[mcn12272-bib-0041] World Health Organization (WHO) (2013) Country implementation of the International Code of Marketing of Breast‐milk Substitutes: status report 2011. World Health Organization: Geneva (revised).

[mcn12272-bib-0042] World Health Organization (WHO) Scientific & Technical Advisory Group (STAG) on Inappropriate Promotion of Foods for Infants and Young Children (2015) Draft clarification and guidance on inappropriate promotion of foods for infants and young children: report of the Scientific and Technical Advisory Group (STAG) on inappropriate promotion of foods for infants and young children, Geneva.

[mcn12272-bib-0043] World Health Organization (WHO) & UNICEF (2003) Global strategy for infant and young child feeding. WHO: Geneva.

